# Immunomodulation of B Lymphocytes by Prebiotics, Probiotics and Synbiotics: Application in Pathologies

**DOI:** 10.3390/nu15020269

**Published:** 2023-01-05

**Authors:** Anaïs Rousseaux, Carole Brosseau, Marie Bodinier

**Affiliations:** INRAE Pays de la Loire, UR1268 BIA, Rue de la Géraudière, BP 71627, CEDEX 01, 44316 Nantes, France

**Keywords:** prebiotic, probiotic, synbiotic, B lymphocyte, immunomodulation

## Abstract

Introduction: Prebiotics, probiotics and synbiotics are known to have major beneficial effects on human health due to their ability to modify the composition and the function of the gut mucosa, the gut microbiota and the immune system. These components largely function in a healthy population throughout different periods of life to confer homeostasis. Indeed, they can modulate the composition of the gut microbiota by increasing bacteria strands that are beneficial for health, such as *Firmicute* and *Bifidobacteria*, and decreasing harmful bacteria, such as *Enteroccocus*. Their immunomodulation properties have been extensively studied in different innate cells (dendritic cells, macrophages, monocytes) and adaptive cells (Th, Treg, B cells). They can confer a protolerogenic environment but also modulate pro-inflammatory responses. Due to all these beneficial effects, these compounds have been investigated to prevent or to treat different diseases, such as cancer, diabetes, allergies, autoimmune diseases, etc. Regarding the literature, the effects of these components on dendritic cells, monocytes and T cells have been studied and presented in a number of reviews, but their impact on B-cell response has been less widely discussed. Conclusions: For the first time, we propose here a review of the literature on the immunomodulation of B-lymphocytes response by prebiotics, probiotics and synbiotics, both in healthy conditions and in pathologies. Discussion: Promising studies have been performed in animal models, highlighting the potential of prebiotics, probiotics and synbiotics intake to treat or to prevent diseases associated with B-cell immunomodulation, but this needs to be validated in humans with a full characterization of B-cell subsets and not only the humoral response.

## 1. Introduction

### 1.1. Probiotics, Prebiotics and Synbiotics: Definitions and Effects

Probiotics have been defined by the Food and Agriculture Organization of the United Nations and the WHO (FAO/WHO) as live microorganisms that, when administered in adequate amounts, confer health benefits. Prebiotic fibers are conventionally known to serve as substrates for probiotic commensal bacteria, stimulating their growth and activity and leading to the release of short-chain fatty acids and other metabolites in the intestinal tract, providing further health benefits [1]. Synbiotics are a combination of prebiotics and probiotics. Synbiotics may increase probiotic survival and ensure persistence of the probiotic strain within the gut [2].

Prebiotics, probiotics and synbiotics are dietary ingredients with the potential to influence health by modifying the mucosal, immune system and gut microbiota composition and function. More specifically, probiotics and prebiotics have the capacity to enhance the epithelial barrier and increase the exclusion of pathogens by competition or adherence inhibition. They can modulate the gut as well as the systemic immune system [3], especially the innate and adaptive immune responses of the host (stimulation of regulatory T and B cells (Treg, Breg), Th1, Th2 and Th17 responses and humoral response) [4]. Probiotics can directly affect all these systems, whereas prebiotics exert indirect effects via the microbiota but also direct effects independently of the microbiota by interacting with several components of the innate and adaptive immune systems [5]. There is increasing evidence that the addition of fermentable fiber to the diet modulates the function and structure of the gut, modifies the production of gut-derived hormones and improves whole-body glucose homeostasis even in the absence of disease [6]. The immuno-regulatory properties of prebiotics, probiotics and synbiotics have been studied on different innate cells (dendritic cells, macrophages, monocytes) and adaptive cells (Th, Treg, B cells). This review focuses specifically on B-cell immunomodulation.

### 1.2. B Lymphocytes: Ontogeny, Function and Localization

B lymphogenesis begins in the bone marrow, where the hematopoietic stem cells undergo rearrangements at the level of the genes coding for the immunoglobulins (Ig) to become immature B cells [7]. Immature B cells migrate from bone marrow into secondary lymphoid organs to finish their maturation. In secondary lymphoid organs, immature B cells go through a transitional stage (transitional B cells) with the expression of IgM and IgD before differentiation onto conventional follicular B cells involved in T-cell-dependent humoral responses or marginal-zone B cells that are involved in T-cell-independent humoral responses. Conventional follicular B cells differentiate into memory B cells or plasma cells after meeting with an antigen. Plasma cells are the effector cells of the humoral response with the production and secretion of antibodies [8].

During humoral immune responses against antigens, immunoglobulins of the IgM, IgD, IgG, IgA and IgE isotypes may be produced, each expressing a unique profile of effector functions, such as the activation of complement or binding to Fc receptors. IgG is the predominant isotype found in the body with the longest serum half-life [9], facilitating the **phagocytic destruction of microorganisms**. It has the highest opsonization and neutralization activities. Four IgG subclasses (IgG1, IgG2, IgG3 and IgG4) have been identified, exhibiting different functional activities: IgG1 and IgG3 antibodies are generally induced in response to protein antigens, whereas IgG2 and IgG4 are associated with polysaccharide antigens. Secreted IgM is the **first antibody transiently increased upon antigen invasion** and is found primarily in the intravascular space, such as in the bloodstream and lymph fluid [10]. It predominates in primary immune responses to most antigens and is the most efficient complement-fixing immunoglobulin. **IgA plays a pivotal role in the immune function of mucous membranes,** such as the gastrointestinal, respiratory and genitourinary tracts [11]. In addition to immune protection from antigens, IgA also has essential anti-inflammatory functions primarily mediated through the induction of immune tolerance via regulatory T cells and dendritic cells. The most prevalent illnesses associated with IgA deficiency are recurrent sino-pulmonary infections but others include allergic diseases and autoimmune conditions. **IgE antibodies are well known for their role in mediating allergic reactions**; however, their role in mammalian evolution appears to be most efficient in defense against parasites and animal venoms [12]. Circulating IgD is found at very low levels in the serum and can react with specific bacterial proteins resulting in B cell stimulation and activation. IgD **acts as an antigen receptor on activated B cells.**

Another B-lymphocyte population has recently been identified in regulatory B cells (Breg). Their origin is disputed in the literature, but they play a crucial role in maintaining the balance of the immune system with the production of anti-inflammatory interleukins, such as IL-10, TGF-β, IL-35 or granzyme B [13]. Among secondary lymphoid organs, gut-associated lymphoid tissue (GALT) is identified as the main secondary lymphoid organ for B-cell development; the Payer’s Patches (PP) in particular [14]. Some studies consider the GALT as a primary lymphoid organ for B-cell development in some species, but this is controversial [15]. Therefore, prebiotics and probiotics, which modify the microbiota, also modulate B-cell response.

This review is the first to compile articles regarding the immunomodulation of B lymphocytes by prebiotics, probiotics and synbiotics and also their application to pathologies associated with B-cell modulation. To select **the literature references, we used the following keywords** on PubMed:

- “prebiotics and B cells”: we found 18 relevant publications in a health context and 14 relevant publications in pathological context.
- “probiotics and B cells”: we found 10 relevant publications in a health context and 10 relevant publications in pathological context.
- “synbiotics and B cells”: we found 3 relevant publications in a health context and 2 relevant publications in pathological context.

By reading these original papers and reviews, we added extra-publications which we thought were relevant to our topic and had not found with the previous keywords. No time frame was selected.

## 2. Immunomodulation of B Lymphocytes by Prebiotics, Probiotics and Synbiotics Supplementation in Healthy Individuals

### 2.1. Effects of Prebiotic Supplementation on B-Cell Immunomodulation (Table 1)

In this review, we identified 25 studies (23 preclinical and 2 clinical studies) studying the effects of prebiotics on the B-cell response. It is important to note that the prebiotic supplementations investigated in these studies were performed at different life stages.

#### 2.1.1. Prebiotic Supplementation during Gestation

Only three preclinical studies investigated the effects of an antenatal prebiotic supplementation on B cells and demonstrated that the immunomodulation of B cells by prebiotics can occur prenatally. Brosseau and colleagues demonstrated that mouse supplementation with GOS/Inulin during gestation in dams increased the frequency of Breg expressing CD9 both in the uterus and in the placenta and Breg expressing CD25 in the placenta, compared to dams fed a controlled diet [16]. The tolerogenic environment present in feto-maternal tissues of prebiotic-supplemented dams was also found in the fetus, characterized by an increased frequency of Breg expressing CD9 in the fetal bone marrow and Breg expressing CD25 in the fetal intestine. CD9^+^ Breg was shown to secrete IL-10, highlighting its immunoregulatory properties. Very interestingly, in the pups at 7 weeks of age, the level of CD9^+^ Bregs was higher in the mesenteric lymph nodes (MLN) of pups issued from prebiotics supplemented dams. It was concluded that prebiotic supplementation during pregnancy leads to the transmission of specific immune factors from mother to child, allowing the establishment of tolerogenic immune imprinting in the fetus. This may be beneficial for infant health outcomes, such as allergy prevention [17]. Still, in the context of the antenatal period, in ovo injection of raffinose was investigated in terms of growth performance in chickens [18]. Even if injection of raffinose had no significant effect on the body weight of chicks, the expression levels of chB6 (which is a biomarker of B cell precursor and throughout the B cell development) were significantly enhanced in the small intestine by high-dose raffinose. This result suggests that the number of total B lymphocytes was increased by antenatal injection of raffinose, which can lead to the development of immunity in the small intestine. **In conclusion, prebiotics administered during gestation can modulate the expression of genes associated with B cells in order to increase their ontogeny in the intestine, which leads to the transmission of specific B immune factors from mother to child, allowing the establishment of tolerogenic B immune imprinting in the fetus and the child.**

#### 2.1.2. Prebiotic Supplementation during Lactation

After birth, the post-natal period is crucial for the maturation of the immune system, largely influenced by diet—especially breastmilk intake. Breastmilk plays a crucial role in the immune system maturation of the child, especially via human milk oligosaccharides (HMOs). Due to their immunoregulatory properties, HMOs are considered as prebiotics naturally produced by the mother [19]. High fucosylation is a general feature of human milk that can be modified by several fucosyltransferases (Fut proteins), including Fut2, Fut3 and Fut8. It has been shown that Fut8^+/−^ maternal mouse-fed neonates had a lower rate of splenic CD19^+^CD69^+^ B lymphocytes and an attenuated humoral immune response, highlighting the key role of the core fucosylation of maternal milk in B cell activation [20]. Because of their properties, prebiotic oligosaccharides have been used in infant formula to mimic HMO. We identified five studies performed on animals and one on humans that mentioned an effect of prebiotic supplementation during lactation on B cells. It was shown that bitches supplemented with fructooligosaccharides (FOS) during gestation exhibit higher colostrum and milk IgM content without an effect on IgG1, IgG2 and IgA level [21]. In a neonatal piglet model used to study the effect of diet in early life (after weaning and over the course of 19 days), the addition of galactooligosaccharides (GOS) into the milk diet increased the IgA levels in the saliva after 19 days of supplementation [22]. In day-old chickens fed with FOS or milk oligosaccharide (MOS)-supplemented seeds, the percentage of total B cells was lower in the cecal tonsil, which is a major GALT, than those in the control group, but the percentage of IgM-, IgG- and IgA-positive cells was similar among the groups, showing that the plasmocyte secretion was maintained [23]. From hatching to 25 days of age, FOS-treated birds had higher plasma antibody titers of both IgM and IgG than in controls, while MOS did not influence plasma antibody concentration. Finally, Nakamura et al. examined whether or not diets enriched with prebiotics during lactation had a beneficial effect on the mucosal immune system [24]. In their study, newborn mice, accompanied by their dams until 21 days of age, were fed either a control diet or a diet supplemented with fructooligosaccharides (FOS). Total IgA levels in the jejunum, ileum and colon of FOS-supplemented mice were about two-fold higher than those in the control group. Moreover, the percentage of B-cell-expressing IgA in PP was significantly higher in the FOS diet group, suggesting that isotype switching from IgM to IgA in PP B cells might be enhanced in vivo by prebiotics in early life. Finally, in humans, Rummens and colleagues conducted a double-blind, randomized placebo-controlled clinical study in order to determine the effect of an infant milk formula supplemented with short-chain (sc) galacto and long-chain (lc) fructooligosaccharides (scGOS/lcFOS) on basal immune parameters in 215 healthy infants over the course of 26 weeks of life [25]. They used a formula-fed group and a breastfed group as controls. They demonstrated that at 8 weeks of age, the percentage of CD23^+^ B cells was decreased in the prebiotic-fed group compared to the breastfed group. CD23 is a low-affinity receptor for IgE that enhances the antibody response, promotes the production of IgE in its soluble form and inhibits the production of membrane IgE [26]. **To sum up, these studies have provided novel evidence for the critical role of HMOs/prebiotics not only in shaping the early-life gut microbiota but also in promoting B cell activation and humoral response at local (gut) and systemic levels of neonates.**

#### 2.1.3. Prebiotic Supplementation during Adulthood

Sixteen studies have investigated the effect of prebiotic supplementation on the modulation of the gut microbiota composition and the modulation of the B cell immune response in adulthood and in different animal models (1 clinical and 15 pre-clinical studies).

Preclinical studies have been performed in dogs, rats and mice, and they focused on B cell humoral response. Field and colleagues conducted a study on adult dogs, demonstrating that adding fermentable fiber (beet pulp, oligofructose powder and gum arabic) to the diet for two weeks can modulate the type and function of cells from different regions of the GALT [27]. Specifically, they found that switching from a low to high fermentable fiber diet resulted in lower mitogen responses in areas involving B cell function (lamina propria and PPs) and a decreased proportion of B cells in the peripheral blood. However, dog supplementation with chicory [28] or inulin [29] did not affect serum concentrations of IgA, IgM, or IgG compared to control dogs. Finally, it was demonstrated that dog supplementation with both FOS and MOS increased ileal IgA concentrations, whereas FOS or MOS alone had no effects [30].Additionally, some studies conducted on rats have investigated the effects of prebiotic supplementation for a few weeks on immunoglobulin secretion by plasma B cells. For example, feeding rats for three weeks with lactulose is associated with increases in IgA secretion or IgA^+^ B cells in GALT [31]. Others showed that rats fed prebiotics (such as pectin, glucomannan, konjak mannan, or chitosan) had significantly higher serum IgA, IgM, and IgG concentrations and a higher frequency of IgA, IgM, and IgG plasma B cells in MLN and in the spleen than those fed control diet [32,33,34]. In the same way, inulin enriched with an oligofructose diet increased the production of secretory IgA in the caecum [35]. Interestingly, serum and MLN IgE concentrations in rats fed konjak mannan, pectin and chitosan for two weeks were significantly lower than in those fed cellulose [32]. It was shown in mice aged 8 weeks that the intake of FOS had no effect on the serum levels of IgA, IgM, and IgG, the frequency of B lymphocytes in the spleen and peripheral blood, and IL-10 secretion [36]. Similar results were found when mice were fed a diet enriched in inulin or oligofructose [37]. However, the levels of fecal IgA were higher in the FOS-supplemented group compared to inulin, oligofructose, or the non-supplemented groups. Supplementation of 8-week-old mice with ***Lycium barbarum*** polysaccharide also increased the levels of IgA in the colon content [38]. It was also demonstrated that FOS supplementation increased the rate of B cells in PP and their IgA secretion in a dose-dependent manner [39,40]. In contrast, FOS suppressed serum IgG1.

Finally, in a clinical trial of humans, xylo-oligosaccharide (XOS) (8g per day), was given to healthy adults (25–65 years) for 21 days [41]. Lower IL-10 production was observed by blood cells stimulated in vitro from XOS-supplemented adults compared to the control group; however, the level of blood B cells (and T cells) was similar.

So, **prebiotic supplementation during adulthood has no major effects on peripheral B cell frequency in humans but increases the frequency of Ig-secreting B cells in secondary lymphoid organs in animal models.**

**Figure 1:**
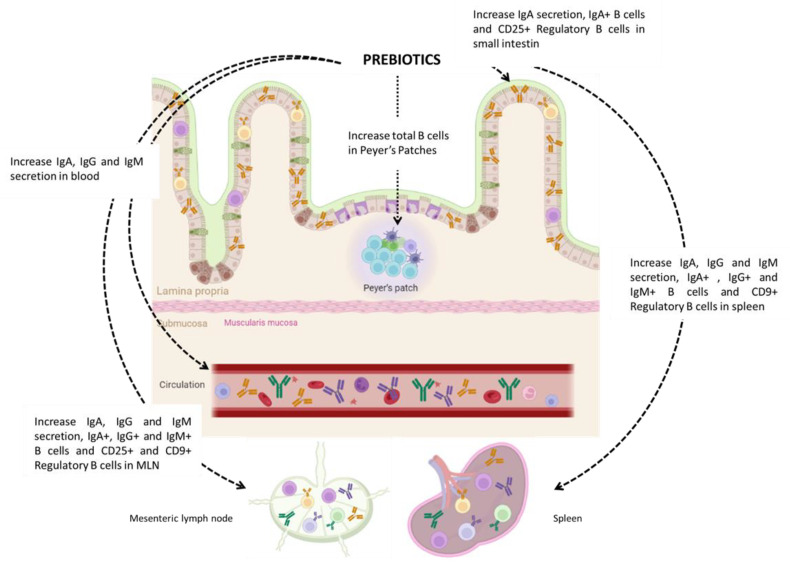

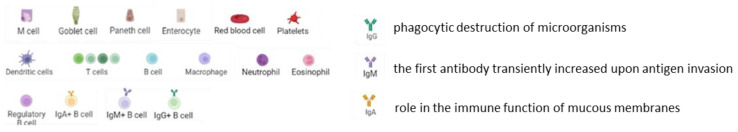
Effects of prebiotics on the immune B response in hosts. Prebiotics boost the immune B response in local and systemic manners. Prebiotcs increase IgA secretion, IgA^+^ B cell number, and regulatory B cells in small intestine. Prebiotics induce a higher percentage of total B cell in Peyer’s Patches and increase IgA, IgG, and IgM secretion in the serum. Prebiotics up-regulate immunoglobulin secretion and IgA^+^, IGM^+^, and IgG^+^ B cells also in mesenteric lymph nodes. Futhermore, prebiotics modulate systemic B response with an increase in Ig secretion and IgA^+^, IGM^+^, and IgG^+^ B cells in the spleen.

**In conclusion, many studies have investigated the effect of prebiotic supplementation on the modulation of the gut microbiota composition and the modulation of the immune system in adulthood and in different models (Table 1, Figure 1). However, only few pre-clinical studies exist that investigate the effects of prebiotics on B cell modulation, and only one was conducted in a human clinical trial. It seems that early prebiotic supplementation modulates B cells both in the GALT and at the periphery, whereas prebiotic supplementation in adults only has an effect locally in the intestine. However, this was only demonstrated in pre-clinical studies. More studies are needed to fully characterize the effects of prebiotics on B cells in humans, especially on regulatory subsets but also on pro-inflammatory B cells and the humoral response.**

Prebiotic supplementation can:

- Increase the expression of genes associated with B cells in utero.
- Increase Breg frequency in the gestational tissues and in the fetus, leading to the establishment of tolerogenic immune imprinting in the child.
- Promote B cell activation in neonates.
- Foster the humoral response.

**Table 1:** Effects of prebiotics on immune B lymphocytes in healthy hosts.

Period of Exposure	Models	Type of Prebiotics	Results	Refences
Gestation	Preclinical study Mice	GOS/Inulin (ratio 9/1) during 4 weeks	Increase CD9^+^, CD25^+^ Breg in dams uterus and placenta and in fetal bone marrow (B CD9^+^) and intestine (B CD25^+^)	Brosseau et al. [16]
			Increase CD9^+^, CD25^+^ Breg in dams and CD9^+^ in pups at 7 weeks of age	Selle et al. [17]
Lactation neonatal period		Core-fucosylated oligosaccharides during 3 weeks	Fut8^+/+^ increase total and activated B cell in spleen and thymus	Li et al. [20]
		FOS (5% of diet) during 5 weeks	Increase total IgA in intestine and increase B cell percentage in PPs	Nakamura et al. [24]
	Preclinical study Dogs	scFOS (1% of diet) during 7 weeks	Increase IgM but not IgG or IgA in colostrum and milk	Adogony et al. [21]
	Preclinical study Neonatal piglets	scGOS (0.8% of diet) during 3 weeks	Increase IgA concentration in saliva	Alizadeh et al. [22]
	Preclinical study Ckicken eggs and chickens	RFO (3 doses: 1.5; 3.0; and 4.5 mg) 1 injection in eggs at day 12	Increase B cell marker ChB6 in small intestine	Berrocoso et al. [18]
		Zinc Bacitracin (ZnB, 0.05 g/kg), FOS (5 g/kg) and MOS (5 g/kg) during 3 weeks	ZnB, or MOS, or FOS: no effect on B cell percentage or Ig^+^ B cells FOS alone: Increase IgM and IgG concentration in plasma	Janardhana et al. [23]
	Clinical trial Infants	scGOS/lcFOS (6 g/L) during the first 6 months of life	No effect on total IgE, IgA, IgM, or IgG concentration but decrease CD23^+^ B cells	Raes et al. [25]
Adulthood	Preclinical study Dogs	Beet pulp, FOS and gum arabic (8.7 g/kg) during 2 weeks	Decrease B cell proportion in peripheral blood and decrease mitogen responses involving B cell function in GALT	Field et al. [27]
		Chicory (1% of diet) or Inulin (0–3% of diet) during 4 weeks	No effect on IgA, IgM, or IgG concentration in the serum	Grieshop et al. [28] Verlinden et al. [29]
		FOS (1 g/kg) or MOS (1 g/kg) or FOS + MOS (1 g/kg) during 2 weeks	Increase IgA concentration in small intestine	Swanson et al. [30]
	Preclinical study Rats	Cellulose and lactilose (5% of diet) during 3 weeks	Increase IgA^+^ B cells and IgA secretion in GALT	Kudoh et al. [31]
		Cellulose, Konjak manna, pectin and chitosan (5 g/100 g of diet) during 2 weeks	Increase IgA, IgM, and IgG concentration in serum, increase IgA^+^, IgM^+^, and IgG^+^ B cells and decrease IgE concentration in MLN and spleen	Lim et al. [32]
		Glucomannan (5% of the diet) during 3 weeks; low or high-fiber diet during 24 weeks	Increase IgA, IgM, and IgG concentration in MLN, spleen and serum	Yamada et al. [33] Zusman et al. [34]
		Raftilose (100 g/kg) during 4 weeks	Increase IgA concentration in caecum	Roller et al. [35]
	Preclinical study Mice	FOS (2.5; 3; 5; 7.5; 10% of diet) during 2 weeks	No effect on IgA, IgM, or IgG concentration, on B cells and nor IL-10 secretion	Delgado et al. [36]
			Increase IgA secretion in dose-dependant way	Hosono et al. [42]
			Increase B cells in PPs what ever the dose	Manhart et al. [40]
		Cellulose, FOS, Inulin (100 g/kg) during 6 weeks	Increase fecal IgA secretion	Buddington et al. [37]
		LBP (0.1 mL/10 g of body weight) during 2 weeks	Increase IgA concentration in the colon	Wei Zhu et al. [38]
	Clinical trial Adults	XOS (8 g/day) during 3 weeks	No effect on blood B cells frequency but decrease IL-10 production	Childs et al. [41]

### 2.2. Effects of Probiotic Supplementation on B Cells Immunomodulation (Table 2)

We identified 13 studies (7 preclinical and 6 clinical) reporting the effects of probiotics on B cells, mainly concerning *Lactobacillus rhamnosus* LGG.

#### 2.2.1. *Lactobacillus rhamnosus* LGG

Among lactic acid bacteria, *Lactobacillus rhamnosus* LGG is the most widely tested probiotic, with more than 500 studies and 5 studies specifically investigating the B cell responses. LGG acts on the mucosa of the gastrointestinal tract. As explained above, bone marrow is the major primary lymphoid organ of B lymphogenesis for mammals, but the maturation and activation of B cells occurs in secondary lymphoid organs such as GALT. Shi et al. investigated the effects of ***Lactobacillus rhamnosus*** LGG on the development of B cells in GALT of mice [43]. One-week-old mice were orally administrated with LGG every day for 2 weeks or with PBS as the control. The percentage of pro-B cells, pre-B cells and immature B cells in bone marrow was significantly lower in the LGG group than that of control group. However, the rate of mature B cells in the LGG group was higher compared to the control group, indicating that LGG promotes the development of pro-B to mature B in bone marrow. Similar results were obtained in the intestinal lamina propria. However, in secondary lymphoid organs, LGG had no effects on B-lymphocyte progression in PPs, except for a small increase in pre-B cell rate. In the spleen, the percentage of mature B cells for LGG group increased, whereas in mesenteric lymph nodes, the percentage of mature B cells for the LGG group decreased compared to the control group. Moreover, LGG inhibited the expression of CD40, CD80, and MHC-II on the B cell surface at the beginning of the supplementation but increased the expression of these markers after few days. The authors suggested that LGG intake inhibits B cell activation and antigen-presenting ability to improve conditions for LGG colonization at first, whereas the stable colonization of LGG in the intestine can promote B-lymphocyte activation and antigen-presenting capability. Finally, at the beginning of LGG supplementation, surprisingly the secretion of IgA in serum of LGG group decreased, while the secretion of IgG and IgM increased compared with the non-supplemented group. With the suspension of supplementation, the secretion of IgA and IgM in the serum of the LGG group increased compared with the control group, while the secretion of IgG decreased. To sum up, LGG intervention can promote the development and maturation of B cells, enhance the activation and antigen-presentation ability of B cells, and regulate their Ig secretion.

In the same way, Jin and colleagues investigated the relationship between the gut microbiota especially LGG and the development of B cells in the GALT of piglets [44]. They demonstrated the presence of an early B cell lineage (expressing *MHC-II*, *CD45RC*, *CD172a*, and *RAG2*) in the lamina propria in pigs at different ages. They showed that LGG supplementation promotes the development of the early B lineage, affects the composition of the Ig repertoires of B lymphocytes, and increases the level of IgA in the lamina propria. Moreover, they demonstrated in vitro that the p40 protein derived from LGG can activate the EGFR/AKT and NF-κB signaling pathways, inducing intestinal epithelial cells to secrete a proliferation-inducing ligand (APRIL), which promotes IgA production in B cells. **In conclusion, this study highlighted in piglets the potential of LGG to promote the development of early B lineage and to modulate the humoral response.**

Finally, in humans, based on the hypothesis that LGG would elicit gene expression responses related to B cell activation in jejunal tissue shortly after consumption, a clinical trial was performed on 27 volunteers who ingested LGG and had a jejunum biopsy 2 h after ingestion for RNA sequencing [45]. Clustering analysis revealed that 30% of subjects exhibited a strong and persistent LGG response involving upregulation of genes related to B cell activation through the B cell receptor, including *CD22*, *CD19*, *CD21*, *CD79a*, *CD79B*, and *FCGR2B* genes. Moreover, additional analysis did not show evidence of B cell influx, demonstrating that this B cell signature was likely due to the activation and proliferation of existing B cells rather than B cell migration to the tissue. In conclusion, this study demonstrated the strong gene expression response of gut cells in the jejunum two hours after the ingestion of LGG dominated by B cell activation. These results cannot ascertain whether the LGG-induced B cell activation response is beneficial, but because administration of LGG increased humoral B cell response, this could have beneficial effects in the context of immunization.

**To sum up, LGG intake can modulate gene expression associated with B cell activation, promote the development and maturation of B cells, enhance the activation and antigen-presentation ability of B cells and regulate their immunoglobulin secretion, such as increased IgA secretion involved in tolerance** [46].

#### 2.2.2. *Lactobacillus acidophilus*

One clinical trial aimed to assess innate immune function following a 6-month supplementation from birth with *L. acidophilus* LAVRI-A1 in children at risk of developing allergies. However, no difference was observed in the number of total blood B cells in infants supplemented compared to infants that received a placebo, nor the expression of human leucocyte antigen-DR (HLA-DR) on B cells [47]. **In conclusion, *L. acidophilus* has no effect on B cell number or HLA-DR expression on their surface.**

#### 2.2.3. *Bacillus polyfermenticus* (Bispan)

Probiotic *Bacillus polyfermenticus*, also called the Bispan strain, was used for the treatment of long-term intestinal disorders. Only one clinical study observed the effect of Bispan on B immune response. Kim et al. aimed to evaluate the effect of *B. polyfermenticus* on the immune response of 25 heathy men [48]. The humoral immune response was measured by the number of total B cells and serum level of IgG, IgA and IgM. Total IgG was increased significantly in the Bispan group after 8 weeks of supplementation while serum levels of IgA and IgM were similar between the supplemented and non-supplemented groups. The frequency of B cell was also the same in the two groups. These results suggest that the intake of ***B. polyfermenticus* has a potentially positive effect on immune function by enhancing IgG secretion**, **and this could be interesting in the context of vaccination**.

#### 2.2.4. *Limosilactobacillus reuteri*

*Limosilactobacillus reuteri* is known to have anti-inflammatory effect [49], but only one pre-clinical study observed these effects on the B cell response. Because intestinal PPs form unique niches for bacteria–immune cell interactions which direct host immunity and shape the microbiome, Liu et al. investigated how the oral administration of the probiotic *Limosilactobacillus reuteri* R2LC affects B cells and IgA production in the PPs, as well as the downstream consequences on intestinal microbiota and susceptibility to inflammation [42]. First, they identified two distinct B cell subsets in mice based on size, phenotype and function: (1) small cells, known as pre-germinal center-like B cells, IgD^+^/GL7^−^/S1PR1^+^/Bcl6, CCR6-expressing B cells, which exhibit bacterial defense properties, and (2) large cells named germinal center-like B cells, GL7^+^/S1PR1^−^/Ki67^+^/Bcl6, CD69-expressing B cells, which display intense metabolic activity. Peroral *L. reuteri* supplementation expanded both B cell subsets, leading to an increase in the total B cell population from 60 to 70% of total PPs cells. It is notable that the B cell population was not altered in mesenteric lymph nodes. Moreover, *L. reuteri* supplementation enhanced the innate properties of pre-GC-like B cells while retaining them in the sub-epithelial compartment. Furthermore, *L. reuteri* promoted germinal center-like B cell differentiation, which involved the expansion of the germinal center area and autocrine TGFβ-1 activation. Finally, *L. reuteri* promotes B cell IgA responses. The authors concluded that the **PPs sense, enhance and transmit probiotic signals by increasing the numbers and effector functions of distinct B cell subsets, resulting in increased IgA production, modified intestinal microbiota and protection against inflammation.**

#### 2.2.5. *Tetragenococcus halophilus*

*Tetragenococcus halophilus* is a halophilic lactic acid bacterium that is present in miso-a fermented soy paste from Japan. Miso has been shown to have beneficial effects in human health, lowering the risk of cancer, hypertension, inflammation, and lifestyle-related diseases and preventing aging [50]. One preclinical and one clinical study used *Tetragenococcus halophilus* as the probiotic in order to observe its effect on B lymphocytes. A study conducted by Kumazawa and colleagues evaluated the immunoregulatory functions of *T. halophilus* species isolated from miso [51]. They screened 56 strains that facilitated the upregulation of activation markers such as CD86 on B cells in vitro. Of these, seven strains were found to preferentially induce the CD86 expression on B cells. Furthermore, DNA microarray analysis revealed that one specific *T. halophilus* strain significantly increased the gene expressions of *CD86*, *CD70*, *IL-10*, *INF-γ* and *IL-22* in B cells. Mice fed a diet containing 1% of this strain of *T. halophilus* exhibited significantly greater IgA production in the serum. Furthermore, this diet augmented the ovalbumin-specific IgG titer in mice upon ovalbumin immunization. Thus, they demonstrated that ***T. halophilus* is a strong immunomodulatory strain of B cells.**

#### 2.2.6. *Bifidobacteria*

*Bifidobacteria* are the most common species of bacteria within normal human gut microbiota. They are particularly common in newborns. *Bifidobacteria* have numerous beneficial effects on human health, including the regulation of digestion; the prevention of infections with enteric pathogens; the modulation of the immune system; and the inhibition of colon cancer development, anti-allergic properties, and protective effects against acute diarrhea [52]. However, the effects of *bifidobacteria* on B lymphocytes have only been investigated in one preclinical study conducted in mice. They investigated the effects of two concentrations of *bifidobacteria* on immune responses by measuring the levels of polyclonal immunoglobulins in serum samples [53]. Immunoglobulins IgG, IgM and IgA showed a significantly higher rate in supplemented-diet mice compared to the control, highlighting **the effects of** ***bifidobacteria*** **on humoral immunity**.

#### 2.2.7. Mixture of Probiotics

Using mixture of probiotics also makes it possible to obtain broader beneficial effects on the host but can also have more significant effects in terms of the modification of the microbiota and the stimulation of the immune system. Prebiotic mixtures often comprise *Bifidobacteria* and *Lactobacillus* strains, since their benefic effects have been demonstrated in preclinical and clinical studies. However, only two preclinical and one clinical study observed the effect of prebiotic mixtures on the B cell responses. Growing evidence suggests that probiotics may have positive benefits on immune responses following endurance exercise. In this context, forty-one healthy sedentary males were recruited and randomized into four groups: placebo, probiotics (*L. acidophilus*, *L. lactis*, *L. casei*, *B. longum*, *B. bifidum* and *B. infantis*), circuit training with placebo and circuit training with probiotics [54]. Although 12 weeks of circuit training enhanced immune cell count in the blood, **probiotics had no effect on B**-**lymphocyte cell count.**

The study of Andreeva et al. investigated the immune status of calves during the pre-weaning period in association with Vetosporin Zh (*B. subtilis* 12B and *B. subtilis* 11B), Normosil (*Lactobacillus brevis*, *L. plantarum*, *L. acidophilus*, *E. faecium*) and Gumi-malysh [55]. The combination of Vetosporin Zh probiotic with Gumi-malysh for 30 days in one-month-old calves **increased the number of B cells in the blood and IgA and IgG serum production while reducing IgM levels.** However, the results enabled the conclusion that the use of Normosil and Vetosporin Zh probiotics, or the combination of Vetosporin Zh with Gumi-malysh, has a beneficial effect on the immunobiological status of the calves during the pre-weaning period.

Pre-weaning piglet mortality ranges from 5% to 35%, and the major cause is due to the late maturation of the immune system and dietary changes in post-weaned piglets. Hence, a study evaluated the modification of humoral response in the intestine after dietary supplementation of probiotics (*Lactobacillus acidophilus*, *Lactobacillus rhamnosus* and *Bifidobacterium longum*) and zinc in pre- and post-weaned piglets [56]. The IgA^+^ and IgM^+^ B lymphocyte rate was higher in the probiotic-supplemented piglets than in the control group, irrespective of segments of intestine and age group. The authors concluded that the dietary supplementation of **probiotic and zinc was positive as they are able to stimulate immune response in piglets** during the critical early post-weaning period.

**Figure 2:**
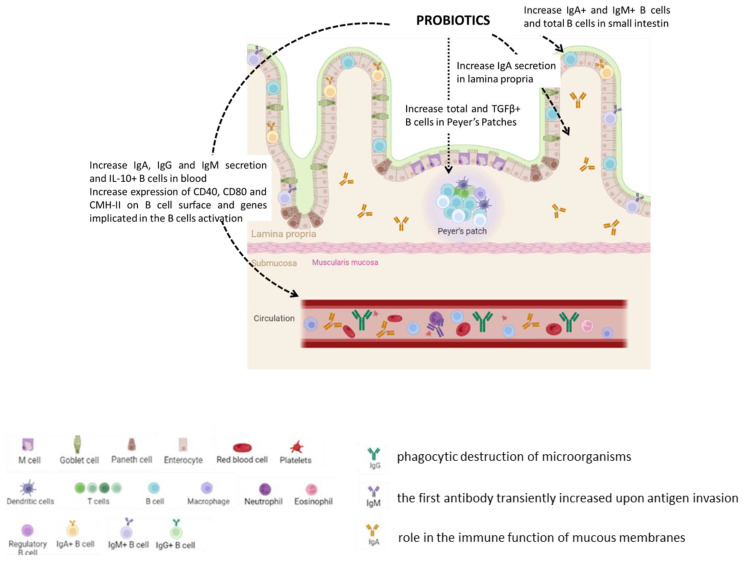
Effects of probiotics on the immune B response in host. Probiotics boost the immune B response in a local manner. Probiotics increase total IgA^+^ and IgM^+^ B cell numbers in small intestine and increase IgA secretion in the lamina propria. Probiotics induce a higher percentage of total and TGFβ^+^ B cell in Peyer’s Patches and increase IgA, IgG and IgM secretion and IL-10^+^ B cell frequency in the serum. To conclude, probiotics induce a higher expression of D40, CD80 and CMH-II on B cell surface and increase genes included in B cell activation in the circulating B cells.

**In conclusion, many studies have investigated the effects of a mixture of probiotics on the stimulation of the immune system but very few have characterized the B cell response (Table 2, Figure 2). The mixture of prebiotics administered in animals induced the B cell rate in intestine and blood and induced the humoral response, but the only studies performed in humans showed no effect from the intake of a probiotic combination on B cell rate. There is clearly a lack of clinical studies that confirm the effects of a supplementation with probiotics on B cells in humans, and the cellular response has yet to be still deciphered.**

- Prebiotic supplementation can increase the IgA and IgG humoral response.
- The cellular response and, in particular, regulatory B cells, have not been observed.

### 2.3. Effects of Synbiotic Supplementation on B Cells Immunomodulation (Table 2)

The effect of synbiotic supplementation on B cells has only been studied in two preclinical and one clinical study. The purpose of the study by Madeg et al. was to determine how pre- and synbiotic administration in ovo into the air chamber influenced the immune-cell composition and localization in the ileum, cecal tonsils and bursa of the Fabricius of broilers [57]. Hatching eggs were treated with a prebiotic (inulin or Bi2 tos^®^) or synbiotic (composed of inulin and *Lactococcus lactis* subsp. *lactis* IBB SL1 or Bi2 tos and *Lactococcus lactis* subsp. *cremoris* IBB SC1) or with physiological saline as a control group. The temporary decrease in B cell number in bursa on day 7 after hatching suggested an increased colonization rate of the peripheral lymphoid organs by these cells after inulin, Bi2 tos and synbiotic Bi2 tos- IBB SC1 treatment. In cecal tonsils, the more potent colonization of the GALT by B cells was observed in both synbiotic-treated groups compared with controls. Then, on day 21, in both synbiotic-treated groups, a faster colonization of the cecal tonsils by B cells was detected. Finally, in 21-day-old chickens, the colonization by B cells was higher in the synbiotic Bi2 tos IBB SC1 than in the Bi2 tos group. The authors concluded that **prebiotics and particularly synbiotics administrated in ovo stimulated B cell development and colonization in the GALT after hatching.**

Another study investigated the effects of administration of *Lactobacillus paracasei* and FOS Raftilose P95 on the immune system of piglets 10 days after birth and 10 days after weaning under standard farming conditions [58]. They found an increased **number of CD19^+^ B cells in the blood of synbiotic-fed piglets** compared to probiotic alone and the control group.

In a clinical trial, prebiotic (XOS), probiotic (*Bifidobacterium animalis* subsp. *lactis* Bi-07) and synbiotic was given to healthy adults aged 25–65 years for 21 days in order to determine immune modulations after microbiota modification [41]. They demonstrated that **supplementation with synbiotics resulted in a lower expression of CD19 on B cells, which may be indicative of changes in B cell subsets**.

- Because of the low number of studies in the literature, we cannot draw a conclusion on the effect of synbiotics on the B cell response (Table 2).
- More studies are needed to obtain more information about synbiotics’ mechanisms on the immune B response.

**To conclude, we counted 41 studies in the literature that analyze the effect of prebiotics, probiotics and synbiotics on the B immune response. This is not sufficient to properly reach a conclusion on the effects. Moreover, most of these studies were conducted on animal models, and it is essential to confirm these results in clinical studies. The results demonstrated the effect of prebiotics, probiotics and synbiotics mainly on the maturation and activation of B cells and on the humoral immune response to boost Ig responses (IgA and IgG), and this could have an application in vaccines, in antiviral and antibacterial response, and in the induction of tolerance with microbiota. Some other B cell subsets, such as regulatory B cells, have not yet been investigated.**

**Table 2:** Effects of probiotics and synbiotics on immune B lymphocytes in healthy hosts.

Type of Supplementation	Models	Type of Probiotics or Synbiotics	Results	Refences
Probiotics	Preclinical study mice	*LGG* (10^7^ CFU/10 μL) during 2 weeks	Increase CD40, CD80 and MHC-II expression on B cells and IgG and IgM but decrease IgA secretion	Shi et al. [43]
		*L. reuteri* (10^8^ CFU/100 µL) during 2 weeks	Increase in PP Pre-GC and GC-like B cells, B220+ B cells, expansion of TGFb+ B cells and IgA germline transcript	Liu et al. [42]
		*T. halophilus* (1% of the diet) during 2 weeks	Increase genes of B cell activation, IL-22 and IL-10 induction and IgA and IgG production in B cells, IgA level in feces and serum but no effect on IgG and IgM levels in serum	Kumasawa et al. [51]
		Bifidobacteria (10^8^ CFU/0.5 mL) during 6 weeks	Increase total IgG	El Hadad et al. [53]
	Preclinical study calves	Normosil (30 mL/animal), Vetosporin Zh (20 mL/animal), Gumi-malysh (30 mL/animal) during 3 weeks	Increase number of B cell and IgA and IgG levels in blood and decrease IgM level in serum	Andreeva et al. [55]
	Preclinical study Piglet	*LGG* (10^10^ CFU/5 mL) during 2 weeks	Decrease B cells in lamina propria but increase mature B cells in PPs, IgA leveles in feces and lamina propria	Jin et al. [44]
		*LGG*, *L. acidophilus* and *B. longum*	Increase IgA^+^ and IgM^+^ B cells	Kalita et al. [56]
	Clinical trial Adults	*L. acidophilus*, *L. lactis*, *L. casei*, *B. longum*, *B. bifidum* and *B. infantis* (30^10^ CFU) during 12 weeks	No effect on salivary IgA nor total B cells	Ibrahim et al. [54]
		*LGG* (450 Bn LGG/ 50 mL) 2 h after ingestion	Increase genes of B cell activation (CD22, CD19, CD21, CD79a(IGa) CD79B(IGb), FCGR2B)	Bornholdt et al. [45]
		*Bacillus polyfermenticus* (Bispan) (10^8^ CFU/day) during 8 weeks	No effect on IgA, IgG and IgM levels in serum but increase total IgG	Kim et al. [48]
	Clinical trial Infants	*L. acidophilus* (30^9^ CFU/1–2 mL) during the first 6 months of life	No effect on total B cells	Taylor et al. [47]
Synbiotics	Preclinical study Chickens	Inulin (1.76 mg), Bi2 tos (0.528 mg), *Lactobacillus* (1000 CFU) During 3 weeks	Increase proportion of Bu1 cells in ceacal tonsil but no effect on Bu1+ cell density in cortex and medulla	Madej et al. [57]
	Preclinical Piglets	FOS Raftilose P95 (3 g/day), *Lactobacillus* (10^9^ CFU/g) during 3 weeks	Increase total B cells in blood	Herich et al. [58]
	Clinical trial Adults	XOS (8 g/day), *B. lactis* (10^9^ CFU/day) during 3 weeks	Decrease total B cells but no effect on salivary and fecal IgA concentration	Childs et al. [41]

## 3. Immunomodulation of B Lymphocytes by Prebiotics, Probiotics and Synbiotics Supplementation in Pathological Contexts

Given the data in the literature on the effects of prebiotics, probiotics and synbiotics on B cells, some research teams have investigated the effects of these components in vaccination and pathological contexts (Table 3).

### 3.1. Vaccination

There are 12 clinical and preclinical studies that have observed the effect of prebiotic, probiotic or synbiotic supplementation on the boosted vaccine response associated with B cell response.

#### 3.1.1. Vaccination with Prebiotics

Six clinical and three preclinical trials reported effects of the vaccination combined with prebiotics on the B cell. During the neonatal period, but also seen in elderly persons, the immune responses elicited by infectious pathogens and vaccines are not optimal. However, breastfed infants have a lower risk of developing several types of infectious diseases compared to infants consuming a regular infant formula [59]. Indeed, human milk is composed of oligosaccharides, immunoglobulins and bacteria, which can boost the immune system of the child [60]. It was shown in mice that supplementation with scGOS/lcFOS/2′-FL in early life (from pregnancy, birth or weaning) improved influenza-vaccine-specific humoral immunity, which is linked to proper B cell memory development in a gender-specific manner [61]. At baseline, females showed an increased antibody response to influenza vaccination than males. Nevertheless, the increased trivalent influenza vaccine-specific IgG and IgG1 were observed only in male offspring fed a scGOS/lcFOS/2′-FL diet from weaning or from pregnancy compared to the control group. This mixture had no effect on B cell memory response nor naïve B cells, activated follicular B cells, or class-switched follicular B cells subsets, and there were no differences between genders. However, the percentage of CD138^+^ plasma cells was higher in females supplemented with scGOS/lcFOS/2′-FL from weaning than in males. In the same context of influenza vaccination, Xiao and colleagues detected a significantly higher frequency of CD27^+^ memory B cell mesenteric lymph nodes of cGOS/lcFOS/2′FL-fed adult mice relative to control mice, consistent with increased vaccine-specific IgG1 and IgG2a levels in serum and enhanced influenza vaccine responses [62]. However, these effects can primarily be attributed to the addition of 2′FL. Indeed, in another study feeding adult mice only with 2′FL, Xiao and colleagues had the same results as in their study feeding the mice with cGOS/lcFOS/2′FL, with a higher frequency of CD27^+^ memory B cells, increased vaccine-specific IgG1 and IgG2a levels in serum and enhanced influenza vaccine responses [63]. Benyacoub et al. investigated the effect of FOS/inulin on murine response to the *Salmonella* vaccine and evaluated the relevance toward protection against *Salmonella* infection [64]. Their data suggest that the addition of FOS/inulin in the diet one week before immunization stimulates mucosal immunity and improves the efficacy of an oral vaccine. This was associated with *Salmonella*-specific blood IgG and fecal IgA. Finally, seniors aged 65 and older consuming a formula containing FOS demonstrated an enhanced frequency of B cells and an increased influenza vaccine response [65], while raftilose and raftiline prebiotic supplementation in an elderly population had no effects on the immune response regarding the influenza and pneumococcal vaccination [66]. Finally, previous dietary intervention studies using scGOS/lcFOS did not identify beneficial effects on the development of vaccine-specific antibody responses for hepatitis B [67], hemophilus influenza type b (Hib), tetanus [68], diphtheria, whooping cough or the poliomyelitis [69].

To sum up, vaccination combined with prebiotics is promising in animal models, but only for some vaccinations, and results are not currently conclusive in humans.

#### 3.1.2. Vaccination with Probiotics

Two preclinical studies and one clinical trial investigated the potential of probiotics to increase vaccination efficacy associated with B cell response. Some studies conducted by Kandasamy’s team investigated the beneficial effects of probiotic supplementation during rotavirus vaccination. Indeed, commensals/probiotics modulate mucosal immunity, but the role of early gut-colonizing bacteria in modulating intestinal B cell responses to rotavirus vaccines remains unknown. They co-colonized neonatal gnotobiotic pigs with LGG and *Bifidobacterium animalis lactis* Bb12 to determine their effect on B cell responses to an attenuated human rotavirus Wa strain vaccine [70]. Following retrovirus challenge, probiotic-colonized vaccinated piglets had significantly lower fecal scores and reduced retrovirus shedding titers compared to uncolonized vaccinated pigs. The reduction in diarrhea was significantly correlated with higher intestinal rotavirus IgA antibody titers and intestinal rotavirus-specific IgA antibody-secreting cell numbers. Interestingly, probiotic-colonized vaccinated piglets had significantly higher frequencies of IgA^+^ B cells in the spleen and ileum compared to that of vaccinated piglets. They also showed a significantly higher rate of activated CD21^+^CD2^−^ B cells in the ileum and duodenum. In summary, LGG and *Bifidobacterium animalis lactis* Bb12 beneficially modulated intestinal B cell responses to the rotavirus vaccine. They next wanted to investigate the same combination of probiotics in association with colostrum/milk (mimicking breast/formula-fed infants) on B-lymphocyte responses to the rotavirus vaccine in a neonatal gnotobiotic pig model [71]. In rotavirus-vaccinated pigs, colostrum/milk feeding with probiotic treatment resulted in higher mean serum rotavirus IgA antibody titers and intestinal IgA antibody-secreting cell numbers compared to the colostrum/milk-fed, non-colonized vaccinated pigs. Moreover, colostrum/milk-fed vaccinated pigs with or without probiotics, had lower duodenal and ileal IgG antibody-secreting cell numbers compared to non-colostrum/milk-fed vaccinated pigs, suggesting the suppressive effects of maternal antibodies on intestinal B cells. In humans, similar results were obtained by Isolauri et al., showing enhanced rotavirus IgA responses in LGG-fed infants of unknown milk feeding status after oral immunization with live oral rotavirus vaccine [72]. Thus, these findings suggest that the immunogenicity of oral rotavirus vaccines can safely be increased by simultaneous administration of viable LGG.

**In conclusion, vaccination associated with probiotics is promising regarding the results obtained in animal models especially in the context of rotavirus vaccination associated with an increase in IgA response. However, it is necessary to validate the effect of vaccination combined with probiotics in a clinical trial.**

- In a murine model, prebiotics supplementation improved vaccine-specific humoral immunity linked to proper B cell memory development, but this is not currently conclusive in humans.
- Both in pre-clinical and clinical studies, probiotic intake activated B cell response and improved vaccine-specific humoral immunity.

### 3.2. Pathological Context

In a pathological context, the potential benefit of prebiotic, probiotic and synbiotic supplementation associated with an immune B cell response was investigated in fourteen preclinical studies and three human clinical trials among eight pathologies.

#### 3.2.1. Colorectal Cancer

The disruption of the balance between microbiota and host is related to many diseases, including colorectal cancer. Only one clinical study evaluated the effect of prebiotic supplementation in patients with colorectal cancer. Xie and colleagues investigate the effects of prebiotic (FOS, polydextrose, xylooligosaccharides and resistant dextrin) supplementation on intestinal microbiota structure and immune function in perioperative patients with colorectal cancer [73]. Supplementation with prebiotics significantly increased IgG and IgM rates before surgery. Postoperatively, IgG, IgA and CD19^+^ B cell rates were significantly higher in the prebiotic group. Because previous clinical studies have indicated that natural IgM and IgA antibodies had the potential to induce the apoptosis of tumor cells [74], the authors concluded that **prebiotic intake is recommended seven days before surgery to improve serum immunologic indicators in patients with colorectal cancer**.

#### 3.2.2. Diabetes

Diet is known to modulate the development of diabetes in rats. One preclinical study conducted by Stillie and colleagues determined the effect of an insulin-supplemented diet after weaning on the immune function in diabetic rats and diabetes-resistant rats [75]. Diabetic rats supplemented with inulin had both an increased rate of B lymphocytes in the PPs and a higher number of IgA^+^ B cells in the jejunum. Interestingly, this response was not observed in diabetes-resistant rats. This study demonstrates that diabetic rats have an abnormal response to a normal dietary ingredient when introduced at weaning. **Therefore, prebiotics stimulate B cell proliferation in diabetic individuals in order to dampen the autoimmunity.**

#### 3.2.3. Allergies

Prebiotic, probiotic and synbiotic supplementation was largely investigated to prevent or to treat allergy.

##### Prevention of Allergies

Allergies (cutaneous, food and respiratory) are associated with alterations in the gut microbiota, epithelial barrier and immune tolerance. These dysfunctions are observed within the first months of life, indicating that early interventions are crucial for disease prevention [5].

Two preclinical studies and one clinical study investigated the B cell response associated with the preventive effects of prebiotic supplementation in allergies. As mentioned above, Brosseau and colleagues have demonstrated that mouse prebiotic supplementation with GOS/inulin during gestation induced a tolerogenic environment in gestational tissues and in the fetus associated with increased Breg frequency [16,76]. Very interestingly, these Bregs were also found in the pups at 7 weeks of age. The researchers wanted next to investigate whether these tolerogenic imprintings could prevent allergy occurrence [17]. They demonstrated that GOS/inulin supplementation during gestation protects offspring from wheat food allergy symptoms associated with an increased rate of CD9^+^ Breg and CD25^+^ Breg cells in the lymph nodes of pups issued from prebiotic-supplemented dams. In the same way, Hogenkamp and colleagues investigated the effects of GOS/FOS intake in mice, before and during gestation, on the immune response in the offspring, using a model of allergic asthma [77]. Supplementation during gestation alone induced a reduction of airway allergic symptoms in the offspring. The pups showed higher rates of Treg than control group, but the levels of IgE, IgG1, IgG2a and the immune cell count in bronchoalveolar lavage were similar. However, in a clinical trial, infants at high risk of allergy that received a hypoallergenic whey formula with GOS/FOS showed a reduced incidence of atopic dermatitis associated with a significant reduction in the plasma level of total IgE, IgG1, IgG2 and IgG3, whereas no effect on IgG4 was observed. Cow’s milk protein-specific IgG1 was significantly decreased [78]. This study demonstrated that GOS/FOS supplementation induces a beneficial antibody profile and that the intake of GOS/FOS is a safe method to prevent the progression of allergies throughout the lifespan.

Probiotic supplementation in the context of allergy prevention associated with B cell response was also investigated in one clinical and one preclinical study. Kim and colleagues studied the effect of oral probiotic bacterial administration on the prevention of food allergy in mice [79]. Three-week-old mice were supplemented either with *B. bifidum*, *L. casei* or *E. coli* two weeks before the induction of ovalbumin-induced allergy and until the end of protocol. Only the humoral response was described concerning the B cell response. Probiotic supplementation induced a decrease in OVA-specific IgE and total IgE levels in sera compared the allergic group not supplemented. Interestingly, the concentration of OVA-specific fecal IgA was significantly decreased with the *B. bifidum* and *L. casei* supplementation. The supplementation of *B. bifidum* and *L. casei*, but not *E. coli*, induced a decrease in OVA-specific IgG1 in the serum compared to the allergic group not supplemented. No effect of the different supplementation was observed on the OVA-specific IgG2a or total IgG2a concentration in the serum. The authors concluded that the administration of *E. coli* or *L. casei* decreased the OVA-induced allergy response and could be a useful probiotic bacteria for the prevention of allergy. In a human study, probiotic bacteria (LGG, *L. rhamnosus*, *B. breve* and *Propionibacterium freudenreichii*) or a placebo were given for one month before delivery to mothers and for six months to infants at risk of allergy [80]. Infants supplemented with the probiotic had higher total IgA, IgE and IL-10 levels than infants in the placebo group associated with a decreased risk of eczema and with a decreased risk of allergic disease at an age of 2 years. The findings highlight the impact of chronic microbial exposure as an immune modulator protecting from allergy.

In the context of prevention, the preventive effect of the synbiotic *B. breve* associated with GOS/inulin was investigated in only one murine study [81]. In mice orally sensitized with whey, this synbiotic markedly reduced the allergic skin response and anaphylactic reactions. It did not affect whey-specific IgE and IgG1 responses, but the IgG2a rate was higher, highlighting the potential for dietary intervention with the synbiotic *B. breve* with GOS/inulin in reducing food allergies.

**In conclusion, prebiotic and probiotic supplementation during pregnancy or administered during the first months of life protects children from allergy associated with the establishment of tolerance (increased rate of Breg and Treg cells) and a beneficial humoral response (decrease in specific IgE)**.

##### Treatment of Allergies

Only one murine study assessed the effect of prebiotics supplementation associated with B cell response on the treatment of allergies. The effects of 2′-FL and 6′-sialyllactose (6′-SL) intake on anaphylactic symptoms induced by oral OVA challenge in sensitized mice were investigated [82]. Daily oral supplementation with HMOs attenuated food allergy symptoms. The intake of either 2′FL or 6′-SL had no effect on the rate of total IgE, antigen-specific IgE or antigen-specific IgG1 in OVA-sensitized and OVA-challenged mice. However, 6′-SL supplementation induced a significant increase in OVA-specific IgG2a compared to sensitized mice fed a control diet.

The use of probiotic bacteria to shift mucosal immunity towards Th1 responses supports their use as a viable approach for the treatment of allergic disorders. Many studies have shown the effects of probiotic treatment on the suppression of asthma in mice, associated with decreased frequency of Th1, Th2 and CD8^+^ T cells in the airways compared to control mice [83]. However, B cell subsets have never been investigated except for the humoral immune response with Ig secretion. For example, in allergic mouse models to OVA, the oral intake of killed *L. casei Shirota* was shown to stimulate the secretion of Th1 cytokines, resulting in a decreased level of IgE antibodies specific to OVA. [84]. The oral administration of *Lactobacillus acidophilus*, LGG and *B. animalis* also significantly reduced the secretion of total IgE, OVA-specific IgE and IgG1 in the same mouse model of asthma [85]. Similar results were obtained using ***B. infantis*** to treat asthma in the same mouse model as food allergies [86]. In a mouse model of allergic rhinitis, treatment with nasal drops containing *C. butyricum* efficiently inhibited rhinitis [87]. Very interestingly, epithelial cells primed by exposing to *C. butyricum* in vitro upregulated their IL-10 secretion. Co-culture of naive B cells with *C. butyricum*-primed epithelial cells significantly increased IL-10 expression in the B cells becoming Breg with an immune-suppressive function on CD4^+^ T-cell proliferation. These in vitro observations associated with the increased rate of Breg can explain the efficacy of *C. butyricum* treatment of allergic rhinitis described in vivo.

In clinic, the supplementation with probiotic *L. casei* and *B. lactis* to treat patients with food allergy to cow milk has shown no efficacy and no modification in the frequency of total blood B cells [88]. In the same way, a probiotic drink containing a combination of the probiotics *Lactobacillus paracasei*, *Lactobacillus acidophilus* and *B. animalis* in healthy volunteers and patients with atopic dermatitis demonstrated no effects on B cell frequency nor IgE concentration in serum [89]. However, to investigate the interaction of LGG with skin and gut microbiota and humoral immunity in infants with atopic dermatitis, a clinical trial was performed, supplementing children suffering from atopic dermatitis with LGG in an extensively hydrolyzed casein formula for three months. The proportions of IgA- and IgM-secreting cells decreased significantly in the treated group after 1 month of supplementation [90]. However, at 3 months of LGG supplementation, the number of IgA- and IgM-secreting cells was no longer different between the two groups, but the percentage of CD19^+^CD27^+^ memory immune B cells was increased in the supplemented children compared to the non-supplemented one. In conclusion, LGG supplementation fosters the immune system maturation in children with atopic dermatitis.

The use of the synbiotic FOS/GOS with *B. breve* in the treatment of allergies has shown no effect on B cell modulation or Ig secretion in a mouse model of cow milk allergies [91] nor in children with atopic dermatitis [92].

The therapeutic efficacy of allergen-specific immunotherapy (SIT) on allergic diseases needs to be improved. Shi and colleagues aimed to determine if treatment with *C. butyricum* fosters the effect of SIT on intestinal allergic inflammation [93]. Food allergic mice to OVA were supplemented with SIT or/and *C. butyricum*. The reduction in the intestinal inflammation induced by SIT was significantly promoted with *C. butyricum*. Interestingly, a higher rate of IL-10-producing OVA-specific B cells was observed in mice associated with decreased intestinal allergic inflammation. The same research team aims to promote the effect of SIT on allergic rhinitis by co-administration with *C. butyricum* in human [94]. The results showed that the immunotherapy alone markedly reduced symptoms and medication scores but did not alter the serum-specific IgE and relapsed one month after stopping the immunotherapy. The co-administration of *C. butyricum* significantly promoted the efficacy of the immunotherapy as demonstrated by the suppression of symptoms, medication scores and serum-specific IgE. These effects were maintained during the administration period and even after stopping the treatment. More interestingly, the combination therapy promoted the generation of Breg secreting IL-10 in allergic rhinitis patients. Indeed, before the treatment the rate of peripheral Bregs was lower in allergic rhinitis patients than in the healthy subjects. After 6 months of treatment, the level of Breg was increased in the immunotherapy/*C. butyricum* group, which was maintained throughout the observation period, but the level of Bregs was similar in both the immunotherapy alone group or the *C. butyricum* alone group. Finally, in vitro, they demonstrated the cooperative effect of B cell-receptor activation and butyrate (which is one of the products of *C. butyricum*) on the differential modulation of IgE and IL-10 expression in allergen-specific B cells. They concluded that administration with *C. butyricum* can markedly enhance the efficacy of SIT on allergic rhinitis patients.

**In conclusion, in a murine model, prebiotic intake as a treatment for food allergies reduced the symptoms despite the lack of regulation of the humoral response. Probiotic treatment on the suppression of asthma in mice has shown promising results with reduced symptoms, reduced humoral response and increased rate of Breg cells. However, in human clinical trials, probiotic supplementation as treatment for food allergies or atopic dermatitis had no effects on B cell frequency nor IgE concentration in serum. Interestingly, probiotics can markedly enhance the efficacy of SIT by reducing the symptoms, inducing tolerance with an increased rate of Breg secreting anti-inflammatory cytokines and attenuating the IgE humoral response.**

- B cell response associated with prebiotic intake in the context of allergy was more investigated in prevention than treatment and has shown promising results, especially in the modulation of the humoral response.
- Once again, the humoral response mainly described with a decrease in IgE and the B cellular response needs to be further investigated.
- The use of probiotics has shown major clinical interest in the context of SIT to induce tolerance associated with an increase in Breg rate.

#### 3.2.4. Endotoxemia

The importance of GALT was highlighted not only in intestinal diseases but also in systemic diseases, such as systemic inflammatory response syndrome and sepsis. As mentioned in the introduction, the GALTs, such as PPs, are inductive sites where B lymphocytes are primed against intestinal antigens and subsequently differentiated into memory cells or plasmocytes. PPs are known to be extremely sensitive to stress conditions, including pathophysiologic and dietary stresses. One preclinical study in mice observed the effect of FOS on endotoxemia associated with B cell response. Manhart et al. reported that inducing endotoxemia in mice or feeding them with a protein-deficient diet causes a dramatic atrophy of PP lymphocytes [95]. They next wanted to determine whether FOS exerts an immunomodulating effect on PPs in healthy and endotoxemic mice [40]. They demonstrated that B cell frequency in the PPs was significantly increased in mice supplemented with FOS, both in the healthy and endotoxemic groups. **Then, prebiotics could prevent the diminution of B cells in PPs in the context of endotoxemia.**

#### 3.2.5. Virus Infection

##### COVID-19

Probiotic intake was shown to have beneficial effects during the treatment of COVID-19 by balancing pro-inflammatory and anti-inflammatory cytokine secretion, which are extremely deregulated in COVID-19 patients who suffer from cytokine storms. Li and colleagues performed a single-center retrospective analysis to confirm and explore the possible mechanisms of probiotics in COVID-19 patients [96]. They collected the clinical characteristics, medication and outcomes of 311 severe COVID-19 patients and analyzed the effect of probiotics (combined *Bifidobacterium*, *Lactobacillus*, *Enterococcus* and *Bacillus* tablets; live combined *Bifidobacterium* and *Lactobacillus* tablets and live combined *Bacillus subtilis* and *Enterococcus faecium* enteric-coated capsules) on B cell immunity and inflammation. They found that **probiotics have the potential to mitigate the inflammation associated with an increased frequency of total B lymphocytes and reduced the incidence of secondary infection in COVID-19 patients.**

##### Rotavirus

Rotavirus, a major cause of gastroenteritis in children, induces significant morbidity and mortality in children worldwide. It has been shown that B lymphocytes play a key role in the activation of protective immunity against rotavirus infection. Two studies investigated the effects of prebiotic intake on B cell immuno-modulation in the context of rotavirus infection. One study aimed to evaluate the potential protective role of 2′-FL, GOS/FOS and their combination 2′-FL^+^GOS/FOS on rotavirus-induced diarrhea in suckling rats [97]. The supplementation was performed from the second to the sixteenth day of life by oral gavage, and on day five, a rotavirus strain was orally administered to induce infection. In the assessment of the incidence, severity and duration of diarrhea, both 2′-FL and GOS/FOS showed a beneficial effect in terms of amelioration. The authors demonstrated that 2′-FL induces a significant decrease in IgG2b and IgA in serum and that GOS/FOS supplementation induces a significant decrease in IgG1, IgG2b and IgG2c in serum. The combination of 2′-FL and GOS/FOS showed additive effects in some variables. Similar results were obtained by Morales-Ferré and colleagues that demonstrated that the supplementation with FOS/GOS of rotavirus-infected rats decreased the rate of specific IgA, IgG1 and IgG2a in the serum compared to non-supplemented rats [98]. This decrease in Ig does not appear to be beneficial in the context of virus infection, but this may traduce the regulation of inflammation induced by prebiotics intake once the infection is eliminated and the symptoms have disappeared in order to return to the homeostasis. The **authors concluded that it could be a good strategy to add prebiotics in combination to infant formulas to protect against human rotavirus-induced diarrhea in children.**

Three studies investigated the beneficial effect of probiotic supplementation in the context of rotavirus infection associated with B cell response. The goal of the study by Zhang et al. was to determine the impact of the colonization of gnotobiotic pigs with lactic acid bacteria (*Lactobacillus acidophilus* and *L. reuteri*) on the development of intestinal and systemic B cell responses to human rotavirus [99]. They demonstrated that the rotavirus infection induced similar rotavirus-specific intestinal and systemic antibody and B cell responses in pigs with or without lactic acid bacteria, whereas lactic acid bacteria significantly enhanced total intestinal IgA-secreting cell responses and total serum IgM and intestinal IgM and IgG titers. However, the lactic acid bacteria colonization did not reduce rotavirus shedding or diarrhea. They concluded that these results may be partly due to the short time interval between the first lactic acid bacteria feeding and rotavirus inoculation. Another study conducted by Kandasamy et al. compared the immunomodulatory effects of *Lactobacillus rhamnosus* and ***Escherichia coli*** probiotic bacteria on virulent human rotavirus infection and immunity using neonatal gnotobiotic piglets [70]. They demonstrated that mean peak virus-shedding titers and mean cumulative fecal scores were significantly lower in *Escherichia coli*-colonized samples compared to *Lactobacillus rhamnosus*-colonized or uncolonized piglets. This was correlated with significantly reduced small-intestinal retrovirus IgA antibody responses in *Escherichia coli*-colonized piglets compared to uncolonized piglets. However, the total IgA levels in the intestine were significantly higher in *Escherichia coli*-colonized than in *Lactobacillus rhamnosus*-colonized piglets. Finally, the in vitro treatment of mononuclear cells with these probiotics demonstrated that *Escherichia coli*, but not *Lactobacillus rhamnosus*, induced IL-6, IL-10 and IgA, with the latter partially dependent on IL-10. **These results suggest that these two bacteria strands differentially modulate rotavirus infection and B cell responses.**

Synbiotics may be also a good strategy to treat rotavirus infection. In this view, *Bifidobacterium* species (*B. bifidum* and *B. infantis*), either associated or not with prebiotics (arabino-galactan, iso-malto-dextrins, scFOS), were administered to mice pups to measure their potential synergistic effects on modulating the course of rhesus rotavirus infection, as well as their ability to mediate the associated mucosal and humoral immune responses [100]. The delayed onset and early resolution of diarrhea were observed in *bifidobacteria*-treated, rotavirus-infected mice compared with rotavirus-infected control mice. This was associated with a higher concentration of rotavirus-specific IgA in feces and serum. However, prebiotic supplementation did not shorten the clinical diarrhea course more than that observed with *bifidobacteria* intake alone. In rotavirus-infected mice that were fed with synbiotics, no enhancement of the immune response was found over those treated with the **probiotic** only. The findings suggest that *bifidobacteria* may act as an adjuvant by modulating early mucosal and strong humoral rotavirus-specific immune responses and mitigate the severity of rotavirus-induced diarrhea. However, the prebiotic compounds chosen for this study did not show any synergistic effect with *bifidobacteria*, either in shortening diarrhea or enhancing the immune response. Two other studies conducted in suckling rats investigated the humoral response of a combination of GOS/FOS with *B. breve* on the protection from rotavirus [101]. In Rigo-Adrover’s study, the animals received prebiotic, probiotic or synbiotic intervention from the 3rd to the 21st day of life by oral gavage. Probiotics reduced the incidence, severity and duration of the diarrhea. The prebiotic and synbiotic products improved clinical parameters. Both the prebiotic and the synbiotic, but not the probiotic, significantly reduced viral shedding. At 14 days of life, probiotics had no effects on humoral response, but prebiotic supplementation induces a significant increase in rotavirus-specific IgG and IgM, and an increase in total IgA in the intestinal wash compared to non-supplemented infected rats. Synbiotic supplementation induces an increase in specific IgM in serum compared to non-supplemented infected rats but has no effect on total Ig. At 21 days of life, probiotic supplementation only induced a significant decrease in rotavirus-specific IgA compared to non-supplemented infected rats. Prebiotic supplementation induced a significant increase in rotavirus-specific IgA and IgM as well as a significant decrease in total IgA and IgM in intestinal wash compared to untreated infected groups. Finally, synbiotic supplementation only induces a significant decrease in specific IgG in serum compared to untreated infected rats. In conclusion, supplementation (especially prebiotic) induces protection against viral infection with an increase in rotavirus-specific IgA, IgG and IgM in serum and an increase in total IgA and IgM in the intestine. The authors also demonstrated the efficacy of these intervention in the context of multiple rotavirus infections and modulating the humoral responses [102]. They concluded that a daily intake of the products tested in these preclinical model is highly effective in preventing rotavirus-induced diarrhea, as well as fostering the early B immune response for a future immune response against reinfection. Therefore, **these components may be potential agents for modulating rotavirus infection in infants.**

In conclusion, prebiotic and probiotic intervention has a clinical interest in preventing rotavirus infection, and this is the current the health claim of enriched infant formulas.

#### 3.2.6. Autoimmunity

Only one pre-clinical study based on the effect of oral treatment with probiotic on B cell response in lupus nephritis was performed in the context of autoimmunity. Recent studies have indicated that the dysbiosis of microbiota is related to the onset of systemic lupus erythematosus, such as the down-expression of CD1d and the up-expression of CD86 in B cells. In the study by Li et al., conducted in mice, oral treatment with *Bacteroides fragilis* decreased symptoms of lupus nephritis associated with the decreased serum levels of total IgM and total IgG [103]. Furthermore, they showed that the intake of *B. fragilis* can increase CD1d expression in B cells via the Est-1 pathway, while inhibiting CD86 expression via the SHP-2 signaling pathway to restore the immune response of B lymphocytes in lupus nephritis mice. **This study highlights the potential application of *B. fragilis* to treat manifestations of systemic lupus erythematosus in high-risk individuals.**

#### 3.2.7. Intestinal Inflammation

Only one preclinical study has investigated the effect of probiotic intake on the intestinal inflammation modulated by B cells. In a mouse model of dextran sodium sulfate (DSS)-induced colitis, Liu et al. investigated the effect of *L. reuteri* supplementation on intestinal inflammation [42]. They confirmed the profound inflammation in the colon and also described severe damages in the ileal mucosa and reduced number of B cells (both pre-GC-like B cells and GC-like B cells) in the PPs by more than 50%. Colitis induced significant B-lymphocyte accumulation in MLNs and caused the ileal mucosal infiltration of IgA^+^ plasma cells. The intake of *L. reuteri* for 14 days prevented DSS-induced ileal disruption and attenuated and delayed colitis symptoms. This was associated with preserved PPs integrity, such as the number of both subsets of B cells and reduced B cell accumulation in MLNs. Moreover, *L. reuteri*-supplementation decreased the ileal infiltration of IgA^+^ plasma cells. A strong negative correlation between B lymphocyte numbers in PPs and body weight loss following DSS-treatment was characterized, highlighting that the effects by *L. reuteri* on PPs B cells are essential for colitis prevention. Thus, this study reported that ***L. reuteri*-intake protects against intestinal inflammation by maintaining the functions of PPs, which modifies intestinal IgA production and prevents inflammation-induced microbiota dysbiosis.**

#### 3.2.8. Graft-Versus-Host Disease

Allogeneic hematopoietic stem cell transplantation is used for the treatment of hematologic malignancies, such as leukemia. Although this transplantation is efficient in inducing a response against the malignant cells, it also leads to the development of graft-versus-host disease (GVHD), a severe disease with high mortality and morbidity rates. Therapy for GVHD is commonly based on the nonspecific immunosuppression of the transplanted recipient, resulting in the concomitant inhibition of the transplantation effect. One preclinical study evaluates the potential of probiotic supplementation to avoid GVHD. In their study, Mercadante and colleagues tested in mice an oral treatment of transplant donors with recipient Ags, combined with the intake of *Lactococcus lactis* as a tolerogenic adjuvant to specifically suppress GVHD while sparing hematopoietic stem cell transplantation [104]. They demonstrated that combined therapy administered to the donor mice before the transplant protects the recipient mice from clinical and pathological manifestations of GVHD, resulting in a 100% survival rate. Interestingly, the suppression of B lymphocytes totally abrogated the protection from GVHD. To confirm these results, splenic cells from combined-therapy-treated donors depleted of B cells were reconstituted with purified splenic CD19^+^ B cells from untreated donors. The reconstitution had no effect, and the recipients still developed GVHD. However, protection was completely restored when B cells from a treated donor were used to reconstitute the combined therapy B-depleted spleen. They also highlighted that the protection observed after oral combined therapy administration depends on IL-10-producing Bregs, expressing a high level of CD86 and class II MHC and presenting a phenotype of CD24^high^CD38^high^ transitional cells. Finally, splenic B cells from combined therapy-treated donor mice induce Foxp3^+^ Tregs on recipient mice. **Taken together, these data suggest that combined therapy with recipient Ags, associated with Lactococcus lactis, is a promising strategy for the prevention of GVHD with the preservation of allogeneic hematopoietic stem cell transplantation, offering new opportunities to treat transplanted patients.**

The intake of probiotics and prebiotics to modulate B cell response, and especially the humoral response, to improve the efficacy of SIT and vaccination or to reduce and protect from rotavirus-induced diarrhea are promising in clinic (Table 3).

- However, for other pathologies, more studies are necessary to conclude on the beneficial effect of prebiotics or probiotics.
- Furthermore, several studies (both preclinical and clinical) are needed to understand B-mediated mechanisms and evaluate their effects, not only investigating B cell Ig secretion but also cellular immune response (cytokine secretion, Breg differentiation, etc.).

**Table 3:** Effects of prebiotics, probiotics and synbiotics on the humoral B response in different contexts of pathology.

Pathology	Models	Type of Supplementation	Results	References
**Vaccination**	Preclinical study—Mice	Prebiotics scGOS/lcFOS/2′-FL during 6 weeks—Influenza infection	Increase vaccine specific response with higher IgG1 and IgG2a levels, higher activated B cells (CD27^+^ and CD138^+^ B cells)	Xiao et al. [62]; van den Elsen et al. [61]
		2′-FL (0.25–5% *w*/*w*) during 6 weeks—Influenza infection	Increase vaccine specific response wih higher specific IgG1 and IgG2a in a dose-dependent manner and higher CD27 expression on splenic B-cells	Xiao et al. [63]
		FOS (5% of diet) during 8 weeks—*Salmonella* infection	Increase vaccine specific IgA but no effect on total IgA and IgG	Benyacoub et al. [64]
	Preclinical study—Piglets	Probiotics *LGG* and *B. animalis lactis* (10^5^ CFU) during 5 weeks—Rotavirus infection	Increase vaccine specific response with higher specific IgA and IgA^+^ B cells and less specific IgG	Kandasamy et al. [70]
		*LGG* and *B. lactis* (105 CFU) 2 doses before vaccination—Rotavirus infection	Increase vaccine specific response with higher specific IgA in ileum and duodenum	Chattha et al. [71]
	Clinical trial—Adults	Prebiotics FOS (6 g/day) during 2.5 months—Influenza and Pneumococcal infections	Increase vaccine specific response with higher specific antibody titers	Langkamp-Henken et al. [65]; Bunout et al. [66]
	Clinical trial—Infants	Prebiotics scGOS, lcFOS and pectin derived acidic alogosaccharides (8 g/L) during 12 months—Hepatitis B, Tetanus and Poliomyelitis	No effect on the vaccine specific response against anti-hepatitis B virus, on anti-tetanus, nor the poliomyelitis	Salvini et al. [67]; Stam et al. [68]; van den Berg et al. [69]
		Probiotics *LGG* (30 mL) during the first 5 months of age—Rotavirus infection	Increase IgM sASC against rotavirus but no effect on IgA IgM, and IgG sASC response	Isolauri et al. [72]
**Virus infection**	Preclinical study—Piglets	Probiotics *LAB* (10^3^, 10^4^, 10^5^, 10^6^ CFU) 1 dose at at 3, 5, 7, 9, 11 days of age—Rotavirus infection	No effect on specific IgA, IgG and IgM in serum but increase the developpment of B cell in gut	Zhang et al. [99]
		Probiotics *EcN* (10^5^ CFU) during 5 weeks—Rotavirus infection	Increase total IgA and IgA^+^ B cells in small intestine but decrease specific IgA and IgG antibody titers	Kandasami et al. [70]
	Preclinical study—Mice	Prebiotics scGOS/lcFOS (0.8 g/100 g of body weight) during 2 weeks—Rotavirus infection	Decrease IgG1, IgG2a/b/c and total IgA	Azagra-Boronat et al. [97]
		Probiotics *B. bifidum* (7.5^8^ CFU/mL) and *B. infantis* (7.5^8^ CFU/mL) during 6 weeks—Rotavirus infection	Increase specific IgA response but decrease total and IgA^+^, IgG^+^ B cells	Qiao et al. [100]
	Preclinical study—Rats	*B. breve* (45^8^ CFU/100 g of body weight), scGOS/lcFOS (0.8 g/100 g of body weight) during 3 weeks—Rotavirus infection	Probiotics, prebiotics and synbiotics decrease specific IgA, IgG and IgM in the serum but prebiotics increase specific IgA, IgG and IgM in intestinal wash	Rigo-Adrover et al. [101] Rigo-Adrover et al. [102]
		Synbiotics scGOS/lcFOS (0.8 g/100 g of body weight) and *Lactobifidus* (0.92 g/100 g body weight) during 2 weeks—Rotavirus infection	Decrease the rate of specific IgA, IgG1, IgG2a in the serum	Morales-Ferré et al. [98]
	Clinical trial—Adults	Synbiotics *Bifidobacterium*, *Lactobacillus*, *Enterrococcus* and *Bacillus* (0.5 g/capsule) during 2 weeks—COVID-19 infection	Increase total B lymphocytes	Li et al. [96]
**Allergy**	Preclinical study—Mice	Prevention Prebiotics 4% GOS/Inulin during 5 weeks—Food allergy	Increase CD9^+^ and CD25^+^ Breg	Selle et al. [17]
		Prevention Prebiotics scGOS/lcFOS (3% of the diet) during 2 weeks—Allergic asthma	No effect on IgE, IgG1, IgG2a and on the immune cell count	Hogenkamp et al. [77]
		Prevention Probiotics *B. bifidum*, *L. casei*, *E. coli* (0.2% of the diet) during 8 weeks—Food allergy	Decrease specific and total IgE, specific IgA and IgG1	Kim et al. [79]
		Prevention Synbiotics *B. breve*, GOS and FOS (2% of the diet) during 8 weeks—Food allergy	Increase specific IgA and IgG2a but no effect on specific IgE and IgG1	Schouten et al. [81]
		Treatment Prebiotics 2′-FL and 6′SL (1 mg/200 µL) during 2 weeks—Food allergy	Increase specific IgG2a but no effect on specific and total IgE nor specific IgG1	Castillo-Courtade et al. [82]
		Treatment Prebiotics GOS and FOS (10 mg/kg) or Probiotics *L. acidophilus* (7.5 billion CFU), *LGG* (8.75 billion CFU), and *B. lactis* (8.75 billion CFU) during 1 week—Allergic asthma	Decrease specific and total IgE and IgG1	Whu et al. [85]
		Treatments Probiotcis heat-killed *LcS* (0.05% of the diet) for 2 injections; *B. infantis* (50^10^ CFU/mL) during 2 weeks; *C. butyricum* (50 mg/mL) during 1 week—Allergic asthma, Food allergy	Decrease specific IgE and IgG1 and total IgE	Matsuzaki et al. [84]; Liu et al. [86]; Zeng et al. [87]
		Treatment Synbiotics scGOS, lcFOS (1% of the diet), and *B. breve* (209 CFU/g) during 3 weeks—Food allergy	No effect on specific IgE	van Esch et al. [91]
		AIT Treatment Probiotics *C. butyricum* and *LGG* (10^9^ CFU/500 µL) and SIT (OVA) during 2 weeks—Food allergy	Increase the frequency of IL-10-producing OVA-specific B cell	Shi et al. [93]
	Clinical trial—Infants	Prevention Prebiotics GOS and FOS (8 g/L) during 6 months—Food allergy	Decrease total IgE, IgG1, IgG2 and IgG3 in serum	van Hoffen et al. [78]
		Prevention Probiotics *LGG* (50^9^ CFU), *B. breve* (10^8^ CFU) and *P. shermanii* (20^9^ CFU) during 1 month before delivery to the mother and the first 6 months of age—Allergies	Increase total IgA, IgE	Marschan et al. [80]
		Treatment Probiotics *L. casei* and *B. lactis* during 12 months; Probiotics *LGG* (34^9^ CFU) during 3 months—Food allergy and atopic dermatitis	No effect on mature B cell; decrease IgA and IgM^+^ B cells but increase memory B cells	Hol et al. [88]; Nermes et al. [90]
		Treatment Synbiotics *B. breve* (13^9^ CFU/g), scGOS, and lcFOS (0.8 g/100 mL) during 3 months—Atopic dermatitis	No effect on IgG1/4 concentration	van der Aa et al. [92]
	Clinical trial—Adults	Treatment Probiotics *L. paracasei* (39^8^ CFU/g), *L. acidophilus* (29^4^ CFU/g) and *B. lactis* (59^4^ CFU/g) during 2 months—Atopic dermatitis	No effect on total B cell nor IgE concentration	Roessler et al. [89]
		AIT Treatment Probiotics *C. butyricum* and SIT (Dermatophagoides pteronyssinus (Der p) during 12 months—Allergic rhinitis	Increase frequency of Breg and Derp1 specific B10 cells	Xu et al. [94]
**Diabetes**	Preclinical study—Rats	Prebiotics Inulin (4.8% *w*/*w*) during 2 weeks	Increase proportion on B cells in PP and IgA^+^ B cells in jejunum	Stillie et al. [75]
**Endotoxemia**	Preclinical study—Mice	Prebiotics FOS (10% of the diet) during 2 weeks	Increase B lymphocytes	Manhart et al. [95]
**Autoimmunity**		Probiotics *B. fragilis* (50^8^ CFU/mouse) during 4 weeks	Increase CD1d expression on B cell and decrease CD86	Li et al. [103]
**Intestinal inflammation**		Probiotics *Limosilactobacillus reuteri* (10^8^ CFU/100 µL) during 2 weeks	Increase in PP Pre-GC and GC-like B cells, B220+ B cells, expansion of TGFb+ B cells and IgA germline transcript	Liu et al. [42]
**Graft—versus—host—disease**		Probiotics *L. lactis* during 5 days	Increase IL-10 B cells with higher level of CD86 and MHC-II and increase transitionnal B cells	Mercadante et al. [104]

## 4. Conclusions

Prebiotics, probiotics and synbiotics were investigated for their immune, microbial, physiological and metabolomic properties in healthy individuals, in the prevention of diseases or in the treatment of patients. In the literature, we found 43 publications on prebiotics, 30 publications on probiotics and 11 publications on synbiotics associated with effects on B cell immuno-modulation in healthy and pathological context. Thousands of studies have been performed to characterize the health benefits of prebiotics, probiotics and synbiotics, but only few have observed the B cell response [105,106]. Whereas meta-analyses support the clinical benefits of prebiotics or probiotics intake in populations at the risk of developing diseases (such as patients at risk of developing *Clostridium difficile* associated diarrhea, atopic patients), conflicting results have been reported [45]. Clinical trials examining these compounds are complicated due to inter-individual variability, such as age, antibiotics usage, diet, use of food supplements, underlying disease and by baseline microbiome patterns [107]. The difficulties encountered by studies evaluating synbiotics are mainly the uncoupling of the effects of the prebiotic or probiotic or a synergistic effect of the combined supplement [41].

Promising studies have been performed in animal models, highlighting the potential of prebiotics (17 studies), probiotics (8 studies) and synbiotics (8 studies) intake to treat or to prevent diseases associated with B cell immunomodulation, but this needs to be validated in humans with the full characterization of B cell subsets. Indeed, only 15 clinical studies have investigated the effects of these compounds on the immune modulation of B cells in a pathological context. Moreover, in most studies, the humoral response is well described but the B cell status of differentiation (especially the regulatory response) or cytokine secretion is not. This will help us to understand the mechanism of action of these compounds at the cellular and molecular level.

In healthy individuals, prebiotic and probiotic supplementation impact B cell subsets, modulating gene expression, inducing anti-inflammatory cytokine secretion, promoting their activation and differentiation in Breg and inducing the humoral response. By inducing Breg, prebiotics and probiotics are able to induce tolerance, which may be of great interest to abrogate inflammation in pathologies such as allergies, auto-immunity, colitis and graft-tolerance after transplantation or to promote allergen specific immunotherapy. By inducing B cell activation and the humoral response, prebiotics and probiotics are able to potentiate the inflammatory response toward pathogens and this may be of great interest in the context of vaccination and virus infection.

A rigorous examination of specific and molecular probiotic and prebiotic responses and personalized effects on the intestinal mucosa and its associated immune system in larger human cohorts would be of great value for researchers and healthcare professionals to understand the modes of action and dynamics of probiotics and prebiotics in human health science.
